# MycoBASE: expanding the functional annotation coverage of mycobacterial genomes

**DOI:** 10.1186/s12864-015-2311-9

**Published:** 2015-12-24

**Authors:** Benjamin J. Garcia, Gargi Datta, Rebecca M. Davidson, Michael Strong

**Affiliations:** Computational Bioscience Program, University of Colorado Denver, Anschutz Medical Campus, Aurora, CO USA; Center for Genes, Environment, and Health, National Jewish Health, Denver, CO USA

**Keywords:** Mycobacteria, Annotation, Database

## Abstract

**Background:**

Central to most omic scale experiments is the interpretation and examination of resulting gene lists corresponding to differentially expressed, regulated, or observed gene or protein sets. Complicating interpretation is a lack of functional annotation assigned to a large percentage of many microbial genomes. This is particularly noticeable in mycobacterial genomes, which are significantly divergent from many of the microbial model species used for gene and protein functional characterization, but which are extremely important clinically. Mycobacterial species, ranging from *M. tuberculosis* to *M. abscessus,* are responsible for deadly infectious diseases that kill over 1.5 million people each year across the world. A better understanding of the coding capacity of mycobacterial genomes is therefore necessary to shed increasing light on putative mechanisms of virulence, pathogenesis, and functional adaptations.

**Description:**

Here we describe the improved functional annotation coverage of 11 important mycobacterial genomes, many involved in human diseases including tuberculosis, leprosy, and nontuberculous mycobacterial (NTM) infections. Of the 11 mycobacterial genomes, we provide 9899 new functional annotations, compared to NCBI and TBDB annotations, for genes previously characterized as genes of unknown function, hypothetical, and hypothetical conserved proteins. Functional annotations are available at our newly developed web resource MycoBASE (Mycobacterial Annotation Server) at strong.ucdenver.edu/mycobase.

**Conclusion:**

Improved annotations allow for better understanding and interpretation of genomic and transcriptomic experiments, including analyzing the functional implications of insertions, deletions, and mutations, inferring the function of understudied genes, and determining functional changes resulting from differential expression studies. MycoBASE provides a valuable resource for mycobacterial researchers, through improved and searchable functional annotations and functional enrichment strategies. MycoBASE will be continually supported and updated to include new genomes, enabling a powerful resource to aid the quest to better understand these important pathogenic and environmental species.

## Background

Mycobacterium species represent both environmental and pathogenic organisms that fall into two major groups: tuberculosis complex such as *M. tuberculosis* and *M. bovis* (MTBC), and Non-tuberculous mycobacteria (NTM) such as *M. avium* complex, *M. abscessus* and *M. smegmatis*. It is estimated that across the world 9.6 million people are infected with tuberculosis every year, 3.6 million of these people are not given proper treatment, and 1.5 million people die from infection [1]. NTM infections have become a growing concern as more people with lung infections have positive cultures for NTM species [2], with cystic fibrosis patients representing a disproportionate amount of detected infections [3]. The prevalence of NTM disease, while relatively rare at 86,244 cases in 2010 in the United States [4], is increasing throughout the world [5, 6], with incidence of NTM exceeding that of tuberculosis in the United States [6]. Treatment of NTM disease also presents a problem due to the chronic nature of the disease, antibiotic treatments lasting up to 18 months, and the cost of treatment being higher than that of multi-drug resistant tuberculosis [4]. Better understanding of gene function for these species allows for better interpretation of clinical experiments, leading to an increased understanding of gene roles and potential drug targets.

Predictive functional annotation methods are a standard practice in analyzing genome sequencing data [7]. Current gene annotation and protein functional annotations are the result of both manual curation and prediction based upon machine-learning tools such as GenemarkS [8], RAST [9] and various homology-based methods such as FASTA [10]. Over the past few years, there has been a development of methods that take into account orthology, protein-protein interactions, and text mining, such as eggNOG [11, 12], a tool used to better annotate the M. *tuberculosis* genome. There have also been improvements to homology-based methods, allowing for both improved accuracy and the assigning of GO terms to genes [13]. Improvements in common methodology for annotation prediction has allowed for both better understanding of genomic content and improved analyses performed on genomic and transcriptomic data.

While there are a couple of well curated databases for *M. tuberculosis* data through TBDB [14, 15] and TubercuList [16] and a database devoted to *M. abscessus* in MabsBASE [17], there remains a lack of well-curated databases for mycobacterium genomes as a whole. One early attempt to fill this gap was made by GenoMycDB [18], a collection of six mycobacterial genomes; however, this database has not been updated to include more genomes. TubercuList was later extended into MycoBrowser [19]. This website contains a comprehensive genomic and proteomic database for three additional mycobacterial species; although, it still lacks commonly studied NTM such as *M. avium* complex and *M. abscessus*. While TBDB [14, 15] has grown to include other NTM species, annotations for these species remain limited. PATRIC [20] contains a wide array of annotated genomes, including mycobacteria, however their functional annotations do not perform well for genomes with large amounts of pseudo genes such as *M. leprae*, leading to 3607 extra genes being annotated despite validation of these as pseudogenes [21]. The MycoBASE database was created to extend the functional annotation knowledge of mycobacteria in general, allowing for a better genomic understanding of both a highly prevalent group of infectious agents, tuberculosis complex, and a group of emerging pathogens, NTM.

## Construction and content

### Mycobacteria gene data

Functional reannotation, gene ontology (GO), phage, and transposon annotation was performed on genes of 11 tuberculosis complex and NTM genomes, as shown in Table 1. Predicted open reading frames (ORFs) from these genomes and their functional annotations were downloaded from two different sources: NCBI and TBDB [14, 15] and form the standard for our reannotation efforts.

**Table 1:** Mycobacterial database species

Species	Strain	Abbreviation	Source	Genes	Reference
*M. abscessus sub. abscessus*	ATCC 19977	MAB	TBDB	4942	[40]
*M. tuberculosis*	H37Rv	MTB	NCBI	4018	[16]
*M. bovis*	AF2122/97	MBOVIS	TBDB	3920	[41]
*M. avium*	104	MAV	TBDB	5120	[42]
*M. abscessus sub. massiliense* ^a^	CRM0020	CRM	NCBI	4750	[43]
*M. abscessus sub. massiliense* ^a^	CCUG48898	MMAS	NCBI	5193	[44]
*M. abscessus sub. bolletii*	CIP108541	MBOL	NCBI	4923	[45]
*M. leprae*	TN1	MLEPRAE	TBDB	1605	[21]
*M. intracellulare*	ATCC 13950	MINT	NCBI	5144	[46]
*M. kansasii*	ATCC 12478	MKAN	NCBI	5449	unpublished
*M. smegmatis*	MC2 155	MSMEG	TBTB	6716	unpublished

Species in the initial release of the database that contain both functional and GO term annotation data. ^a^ Subspecies *massiliense* is currently listed as subspecies *bolletii* in NCBI; however, the classification is still being debated and the distinction between *massiliense* and *bolletii* has clinical importance [47]

### Annotation prediction

**Fig. 1:**
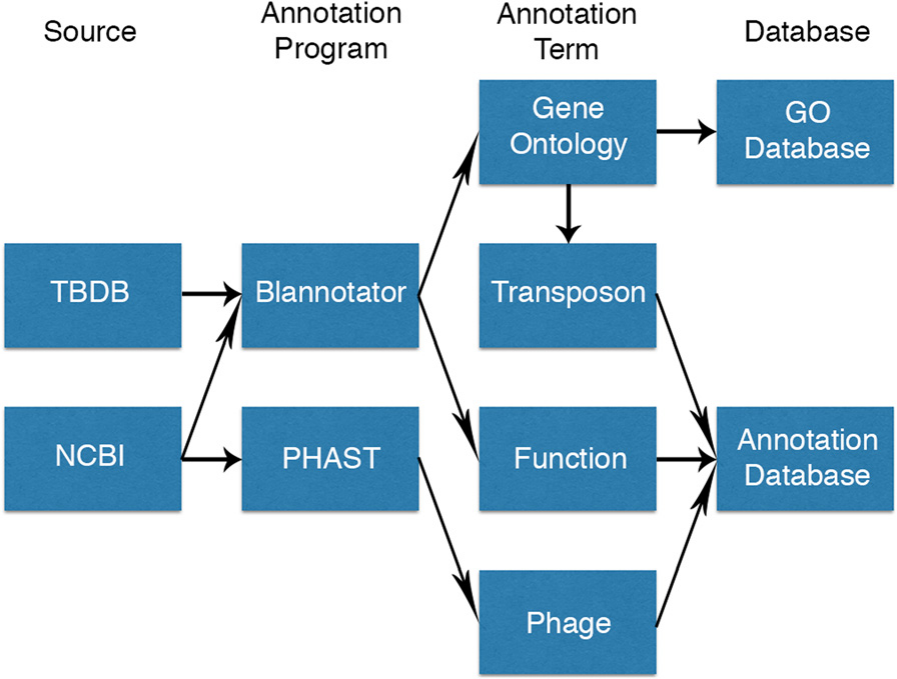
Pipeline for creating improved annotations. Input files were taken from NCBI and TBDB and then annotated using Blannotator and PHAST. The resulting annotations were then used to create databases containing GO terms and a collection of functional, phage, and transposon annotations

Blannotator [13] was chosen for functional reannotation and GO term annotation due to its high accuracy for bacterial genomes relative to other homology-based methods. Blannotator source code [13], UniProt databases [22], GO databases [23], and NCBI-BLAST+ libraries [24] were downloaded onto a linux server. Blannotator source code was then modified to utilize NCBI-BLAST+ libraries and to utilize more threads when running BLAST+. Predicted ORFs from each of the 11 genomes were then translated and annotated with Blannotator (improved annotations). Only the highest scoring functional annotations were used for all downstream assessments and database generation. All GO terms associated with a protein were used due to both the multiple associations between GO terms and a given function, and the hierarchical structure of GO terms. To annotate genes associated with DNA transposons, we used the predicted GO term “transposase activity”. Genes associated with phage regions were annotated with PHAST [25] due to its high accuracy compared to other phage tools. Both phage and transposon are annotated with “YES” or “NA” to denote their likelihood of being part of a phage or transposon region. The output of all of these annotations were then stored in databases described in the Database Structure section with the pipeline for creating these annotations seen in Fig. 1.

### Evaluation of improved functional annotations

For a protein to be considered annotated, the protein must meet at least one of the three characteristics: function, localization, and/or name. Examples of each of the following are as such: “methyltransferase”, “membrane protein”, and “fadE6”. Terms such as “precursor” and “10.1 kDa protein” are excluded, as they do not represent any of these characteristics. Uncharacterized proteins that have protein names are considered annotated as they often make up large families of homologous proteins that have uncertain functions, such as PPE family proteins and UPF/DUF proteins. Uncharacterized proteins where that annotation matches the protein ID are not considered annotations. This annotation guideline conforms to other reannotation ventures such as EggNOG [11], as well as giving consistent coverage of terms across the original and improved annotations.

Preprocessing was performed on annotations to allow for accurate comparison of function, localization, and name. Non-alphanumeric characters were replaced by blanks due to their inconsistent use in separating words, compounding words, and naming of chemical entities. Words that represent homology scoring or redundancy in naming, such as “putative”, “family”, “protein”, etc. are also removed for this evaluation; however, these annotations are maintained in the database.

Overlapping annotations from the original and improved annotations are then compared using bigram Dice’s coefficient, a coefficient used in natural language processing to compare word sets [26]. Bigrams, sets involving two successive letters, are used to preserve some of the lexicon that was eliminated by removing non-alphanumeric characters. Given the structured vocabulary present within both original and improved annotations, Dice’s coefficients offer a precise method for automated comparison of annotations. To identify if two annotations are significantly similar, a Wilcoxin signed rank test with a confidence interval of 95 % was used. The background for the test was the set of Dice’s coefficients generated by comparing original annotations against all other original annotations. An annotation pair between the original and improved was considered significantly similar if their Dice’s coefficient was above the 95 % confidence interval. Reasons for insignificance include: generic annotations, same function but different annotation, similar but different functions, and completely different function.

**Fig. 2:**
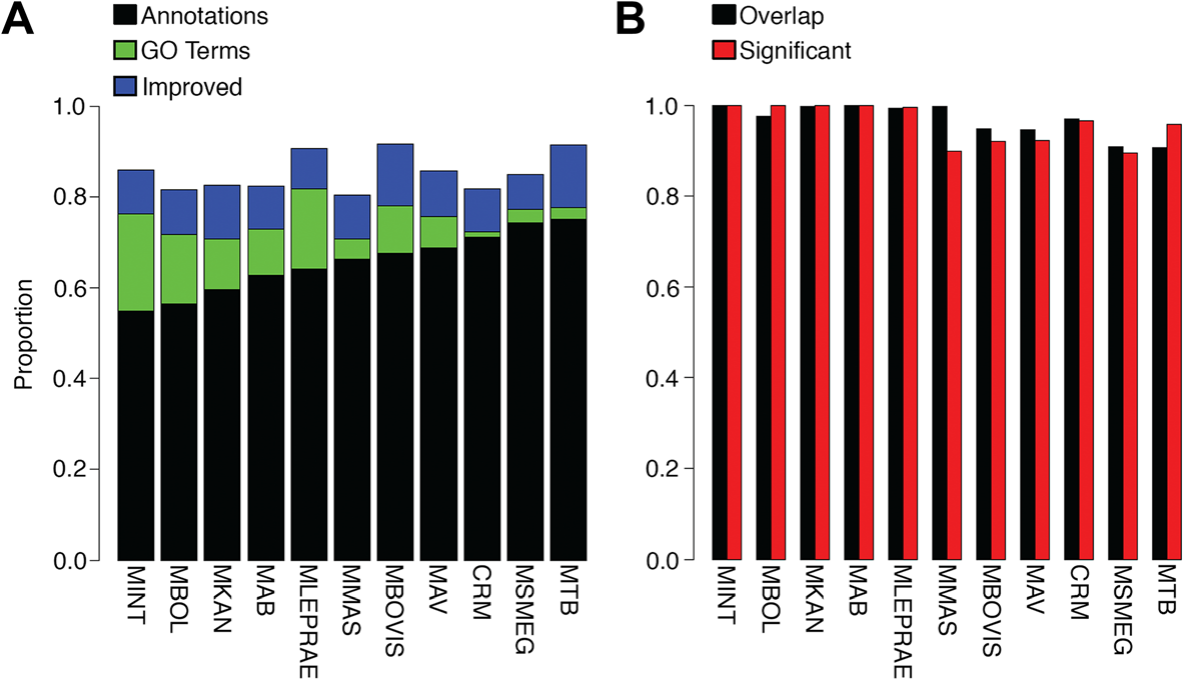
Annotation improvement in the 11 mycobacterial genomes. **a** Proportion of genes with original functional annotations (*black*), GO term annotations (*green*), and improved functional annotations (*blue*). **b** Proportion of original functional annotations with a corresponding improved functional annotation (*black*), and the proportion of these annotations that are more significantly similar than background (*red*)

Each of the 11 Mycobacterial genomes was evaluated for functional annotation and GO term annotation coverage. While none of the 11 genomes had GO terms to evaluate against, all the genomes had functional annotations. Blannotator produced an average increase in functional annotations for each genome of 20 %, shown in Fig. 2a, ranging from 11 % in CRM to 31 % in MINT. In addition to this increase in functional annotations, the average coverage of GO terms was 9 % higher than the original functional annotations. The average coverage for GO terms is 75 % of genes, ranging from 71 % in MMAS to 82 % in MLEPRAE. This results in a significant increase in functional annotations, in addition to having GO terms for functional enrichment testing. Figure 2b shows the percent of annotations that overlapped between the original and improved annotations. This figure also shows the percentage of overlapping annotations that were significantly similar relative to the background. The result of this evaluation showed that an average of 99.6 % of annotations overlapped (range: 97.6 % in MKAN to 100 % in MBOL) and that 93.1 % of these overlapping annotations were significantly similar (range: 89.4 % in CRM to 97 % in MMAS).

### Gene ontology enrichment

Modified one-sided Fisher’s exact tests, similar to those created for EASE scores [27], are used to evaluate enrichment of GO terms in a gene set against a background set. A hypergeometric probability for contingency tables is calculated using an estimation [28], allowing for a more efficient calculator than the direct representation of the Fisher’s exact test. For calculating *p*-values, the GO terms had to meet two criteria: the number of genes associated with the GO term in the gene set is greater than one, and the proportion of genes with the GO term is greater in the gene set than in the background set. All genes in the gene set and the background set, irrespective of whether or not they have GO terms associated with them, count towards the values in the contingency tables. Both the non-multiple testing corrected *p*-values and Bonferroni adjusted *p*-values are ranked and displayed. This Java-based program is available for download and use on the Website.

### Database structure

**Fig. 3:**
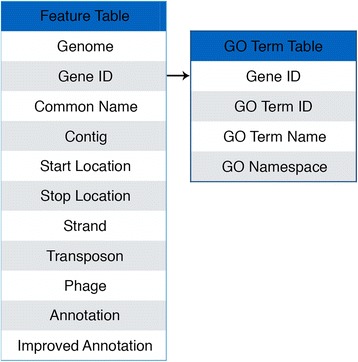
Database structure. Database containing two tables. The first table contains gene features for a given genome. The second table contains GO terms for all genes. These two tables are linked together by gene IDs

The database is made up of two tables, as shown in Fig. 3. The feature table contains all known gene information for a given genome. This contains the strain identifier, the gene ID, the common name from NCBI/TBDB, the location of the gene, whether or not the gene is related to a transposon or phage, the original functional annotation, and the improved functional annotation. This table can be queried by selecting a genome of interest and by either selecting all genes or a supplying a subset of genes. The GO table contains GO information for all genes that contain GO Terms. This GO information contains the ID, the term, and the namespace of a GO term for a given gene. Each gene can contain multiple GO terms. This database is queried through the first table, as these tables are linked together by gene IDs.

### Website

**Fig. 4:**
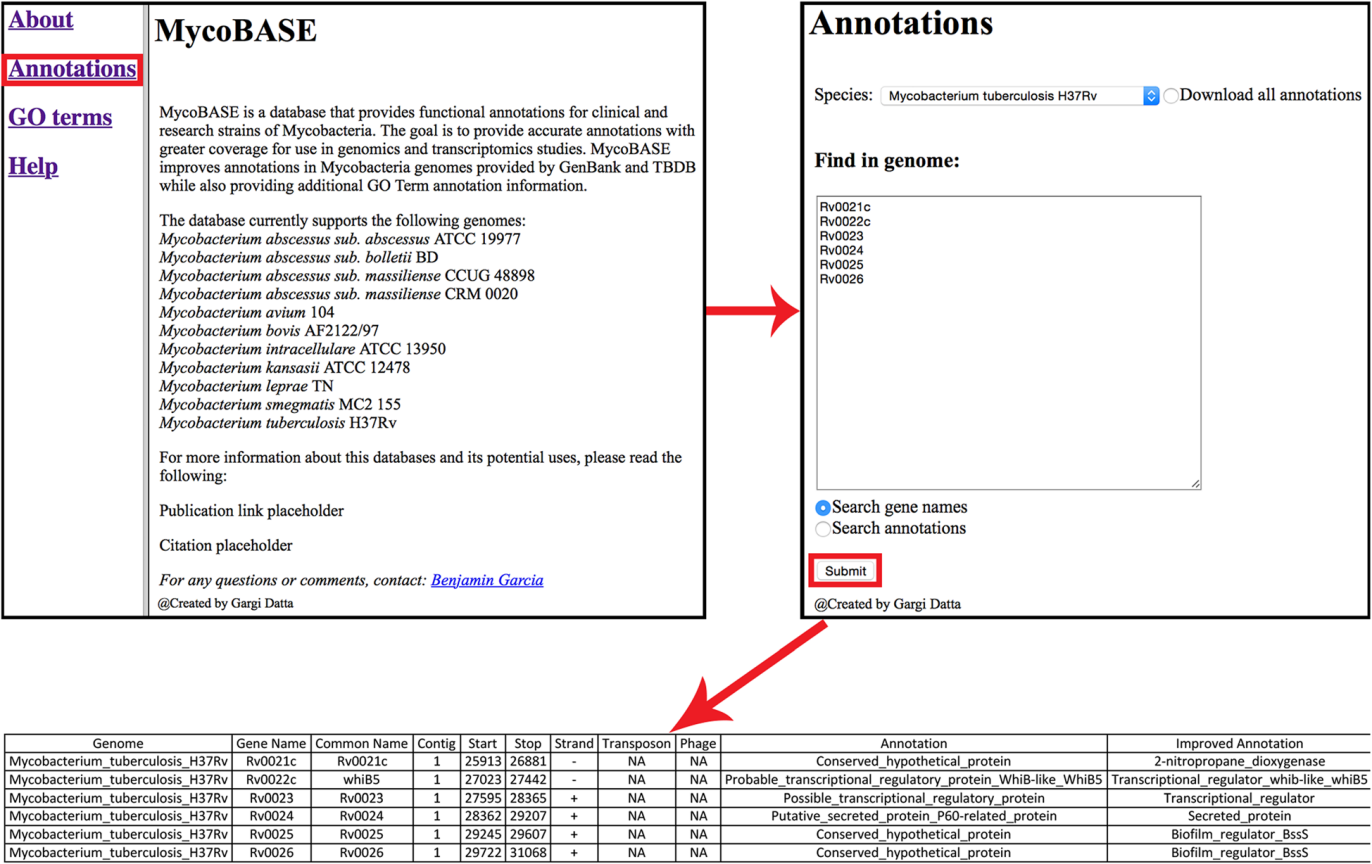
Extracting annotations by gene name from website. To download annotations by gene name first click on the “Annotation” link on the website. Next select your genome of interest from the species dropdown menu. Select the “Search gene names” option button then insert a list of gene names separated by a comma or newline character. Next hit the “Submit” button and the list of annotations associated with the gene names will be downloaded

The database can be accessed from the website: strong.ucdenver.edu/mycobase. From the homepage, users can access pages to search annotations, search GO terms, view a list of currently annotated genomes, and access quick help about using the webpage. On the annotation page, the user first selects their genome of interest from the drop down menu. After selecting the genome of interest, the user selects an option button corresponding to 1) Downloading the whole genome, 2) Searching by gene names, or 3) Searching by annotation. If the user selects search by gene name or annotations, they enter either a single gene/annotation or a list of genes/annotations (separated by comma or newline) into the text box. An example of searching by gene name in the *Mycobacterium tuberculosis* H37Rv genome is “Rv0001”. An example of searching by annotation is “methyltransferase”. Clicking submit downloads a formatted file of genes corresponding to the genome, gene name, or annotation. The header for describing the formatted information is the first line in the file. A simple flow through of downloading annotations corresponding to gene IDs can be seen in Fig. 4.

Searching for GO terms follows a similar format as searching annotations. A user first selects the genome of interest, and then selects the option box associated with either the genome or search by gene id. If searching by gene id, the user inputs either a single gene id or list of gene ids separated by comma or newline. Clicking “submit” downloads the list of GO terms with the first line being the header describing each field. The enrichment program for the modified fisher’s exact test and a use case is included on this page. A description of the required input files and program description are also included in this download. In addition to being able to enrich for GO terms, this program can also enrich for any categorical terms that can meet the input file guidelines, such as other available *M. tuberculosis* categorical terms [29]. Lastly, the help page briefly describes how to download GO Terms and annotations.

## Utility and discussion

### Exploration in genome variability

**Fig. 5:**
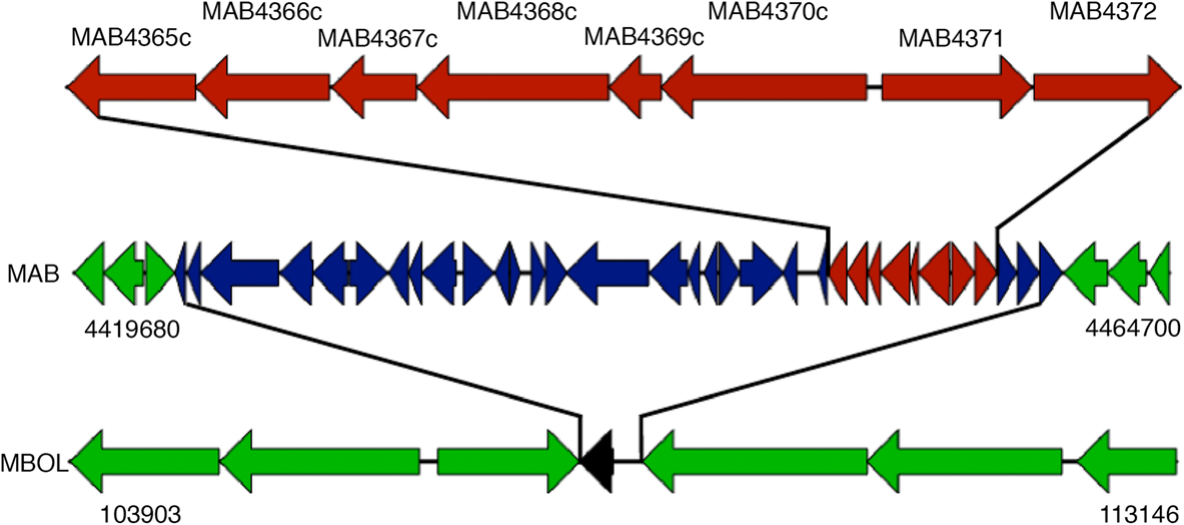
Insertion of gene cassette in *Mycobacterium abscessus* strain ATCC 19977. Insertion of a 37KB insertion region encoding for a cassette of eight biphenyl and aromatic hydrocarbon degradation enzymes (*red arrows*). Conserved genes within MAB and MBOL are shown in *green*, and 34 inserted genes are shown in *blue* and *red*

Predicted genes from MAB and MBOL were compared for sequence homology to differentiate between shared and unique genes between two *Mycobacterium abscessus* genomes. From this analysis we have discovered a 37KB insertion sequence in *Mycobacterium abscessus* ATCC19977, as seen in Fig. 5. Using predicted GO terms for both of these genomes and the Java-based enrichment program, we have found that this insertion sequence contains a cassette of 8 genes associated with biphenyl and aromatic hydrocarbon degradation enzymes, including a group of ferredoxin reductases that are necessary for iron-catalyzed hydroxylation [30, 31]. These enzymes allow for degradation of carbon sources such as plant lignin, crude oil, and natural gases, and environmental contaminants such as petroleum products, PCBs, and PAHs. This degradation activity has been observed in a variety of environmental microbes including mycobacteria [30–34]. This style of analysis has been used to analyze content of deletions in *Mycobacterium abscessus* [35].

### Gene ontology term taxonomy

**Fig. 6:**
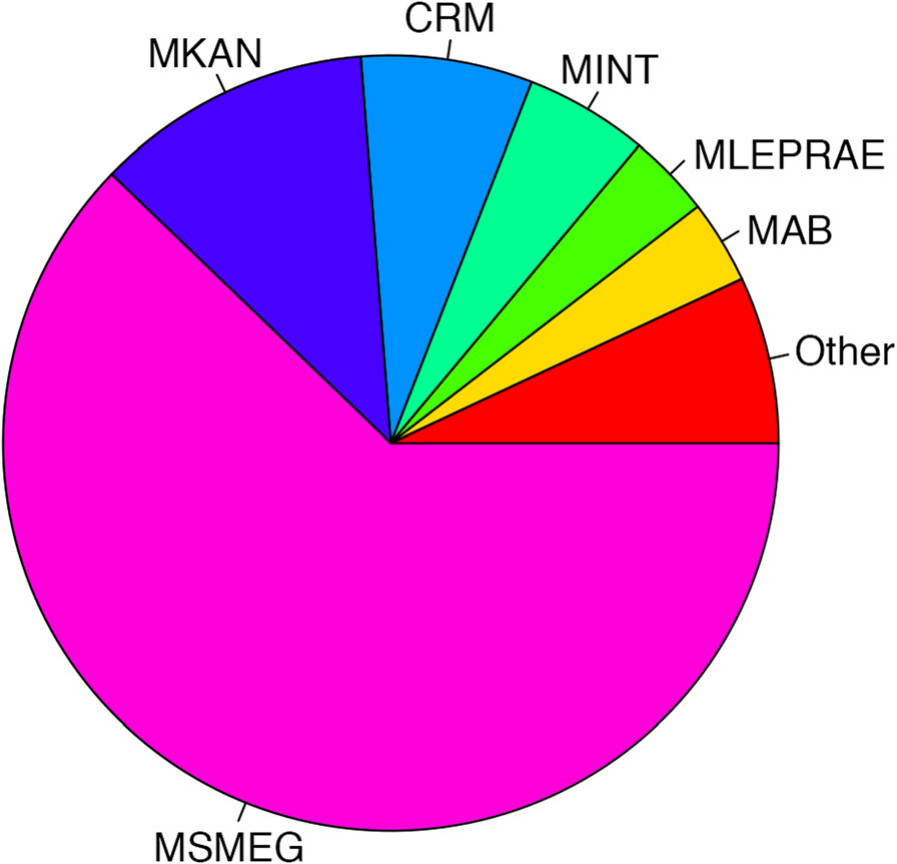
Gene ontology 1 taxa terms. Proportion of the 545 GO terms that are unique to one genome. MSMEG contains the majority of 1 taxa terms, owing to its larger genome and diversity of chemicals that it can metabolize and synthesize

Creating GO term taxonomy allows for identifying both conserved function across multiple mycobacterium and identifying species-specific functions. Slightly more than half of GO terms are shared by the 11 species, with 73 % of terms being shared by more than half of the species, as shown in Table 2. This shows that the majority of function is conserved across mycobacterium species. Of the GO terms associated with MLEPRAE, 91 % of them occur in the 10 other species, suggesting that MLEPRAE contains a fundamental set of functions that define the mycobacterium species. Only 12 % are unique to a single species, with MSMEG accounting for 62 % of one taxa terms (Fig. 6). Much of MSMEG’s unique terms are carbon-based metabolism and synthesis related, suggesting that its larger genome size allows it to both utilize and create additional carbon sources relative to other mycobacterium [36]. However, 61 % of GO terms for MSMEG are still shared across all mycobacterium. While MSMEG has more genes and one taxa GO terms, most of the function within MSMEG is conserved across species suggesting that the increased genome size is due to gene duplication [37]. MTB and MBOVIS share the most 2 taxa terms with 25 % of the total, owing to their similar genome size and their pathogenesis.

**Table 2:** Gene ontology term taxonomy

Taxa	Terms	Percent
1	545	11.8 %
2	171	3.7 %
3	123	2.7 %
4	175	3.8 %
5	210	4.6 %
6	146	3.2 %
7	97	2.1 %
8	89	1.9 %
9	67	1.5 %
10	559	12.1 %
11	2428	52.7 %

Unique GO terms for the intersection of the 11 Mycobacterium species. More than half (52.7 %) of GO terms are shared by all species

### Gene ontology term enrichment between genomes

**Fig. 7:**
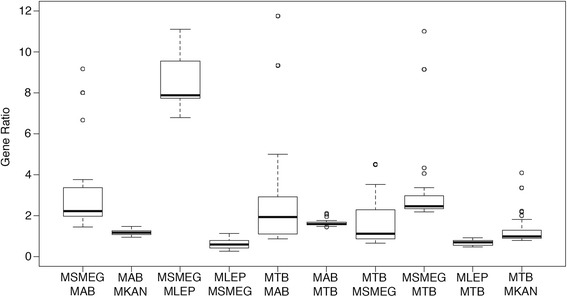
Ratio of genes in enriched GO terms. Plot of the 10 genome pairs with more than one enriched gene. The ratio is the number of genes with a given GO term in one genome over the number of genes with that same GO term in the other genome

To evaluate characteristics of mycobacteria, GO term enrichments were performed on select mycobacteria, as shown in Tables 3 and 4. Backgrounds for these comparisons were the combination of both of the genomes being compared. The *M. abscessus* and *M. tuberculosis* clades acted as controls for the enrichments due to their similar phenotypes and pathogenicity. Upon analysis, there were no enriched GO terms within these sets, affirming the similarity between the genomes and the validity of the method. The other genome pairs represent differences in growth, pathogenicity and clade. Figure 7 shows the ratio of genes associated with enriched GO terms between the genome pairs. While enriched GO terms in MLEPRAE had lower gene ratios than other genomes, there were a higher proportion of these genes in the genome. This suggests that not all GO functions scale with genome size and that genome size differences are an important consideration when performing enrichments.

**Table 3:** GO term enrichment genomes

Genome	Genes	Growth	Type	Clade
MBOV	3920	Slow	Obligate Pathogen	MTBC
MLEP	1605	Slow	Obligate Pathogen	Ungrouped
MTB	4018	Slow	Obligate Pathogen	MTBC
MKAN	5449	Slow	Environmental-Opportunistic Pathogen	Kansasii
CRM	4750	Fast	Environmental-Opportunistic Pathogen	*M. abscessus* group
MAB	4942	Fast	Environmental-Opportunistic Pathogen	*M. abscessus* group
MBOL	4923	Fast	Environmental-Opportunistic Pathogen	*M. abscessus* group
MMAS	5193	Fast	Environmental-Opportunistic Pathogen	*M. abscessus* group
MSMEG	6716	Fast	Environmental	Ungrouped

The number of genes and general phenotypic traits of the genomes used for the comparison

**Table 4:** Gene enrichment comparisons

Genome1	Genome2	Gene difference	Enriched GO terms	Type
MAB	MSMEG	1774	1-35	Different
MAB	MKAN	507	52-1	Different
MSMEG	MLEP	5111	15-270	Different
MTB	MAB	924	48-15	Different
MTB	MSMEG	2698	74-37	Different
MTB	MLEP	2413	0-26	Different
MTB	MKAN	1431	59-0	Different
MAB	MMAS	251	0-0	Same
MAB	MBOL	19	0-0	Same
MAB	CRM	192	0-0	Same
MMAS	MBOL	270	0-0	Same
MTB	MBOV	98	0-0	Same

Both genomes were compared against each other. The gene difference is the difference in the total number of genes between genomes. Enriched GO terms are the number of enriched terms in Genome1 over Genome2 “-” Genome2 over Genome1. If the type is different, then there was either a difference in pathogenicity, slow or fast grower, or the genomes come from different clades

Enrichment of host-pathogen GO terms occurred in all of the pathogen-environment comparisons except MTB-MKAN; however, in this pair, these terms barely failed to meet significance (*p*-value ~0.07), suggesting these terms are still likely an important distinction between the pair. MSMEG had enrichments in carbohydrate transporters over both MAB and MTB. This is related to the fact that MSMEG can metabolize a broader range of carbohydrates relative to other mycobacterium [36] and is supported by the number of one taxa GO terms related to carbon metabolism.

## Conclusion

MycoBASE currently contains 11 mycobacterial genomes with functional and GO term annotations. Our genomes are based off of NCBI gene annotations, allowing for a well-accepted genome leading to consistency across studies. These annotations allow for improved understanding of the genetic content of these genomes, leading to more coverage in analyses involving differential gene content (insertion/deletion of genes, differences in genes across species), genes that are understudied but have homology to genes of known function, and functional analyses of transcriptomics and genomics data using GO terms (the modified Fisher’s program being available for download on our server). These annotations will be available for download, allowing for a wide variety of analyses. Our future plans include adding a greater diversity of genomes to our database, such as *M. africanum* [38], *M. chelonae* [39], and other studied mycobacteria, greatly increasing the number of species in the database.

## Availability and requirements

This database and GO enrichment is available for academic and other non-commercial uses at the website: strong.ucdenver.edu/mycobase.

## Abbreviations

GO: Gene Ontology
MAB: *M. abscessus sub. abscessus* ATCC19977
MTB: *M. tuberculosis* H37Rv
MBOVIS: *M. bovis* AF2122/97
MAV: *M. avium* 104
CRM: *M. abscessus sub. massiliense* CRM0020
MMAS: *M. abscessus sub. massiliense* CCUG48898
MBOL: *M. abscessus sub. bolletii* CIP108541
MLEPRAE: *M. leprae* TN1
MINT: *M. intracellulare* ATCC13950
MKAN: *M. kansasii* ATCC12478
MSMEG: *M. smegmatis* MC2 155
